# ILAE Genetics Literacy series: Progressive myoclonus epilepsies

**DOI:** 10.1002/epd2.20152

**Published:** 2023-09-06

**Authors:** Jillian M. Cameron, Colin A. Ellis, Samuel F. Berkovic, for the ILAE Genetics Commission, the ILAE Genetic Literacy Task Force, Piero Perucca, J. Helen Cross, Holger Lerche, Alina I. Esterhuizen, Iscia Lopes‐Cendes, Meng‐Han Tsai, Daniel H. Lowenstein, Nigel C. K. Tan, Ingo Helbig, Heather C. Mefford, Andreas Brunklaus, Gaetan Lesca, Elizabeth Emma Palmer, Amy McTague, Faiza Fakhfakh, Norman Delanty, Daniel H. Lowenstein, Nigel C. K. Tan, Alina I. Esterhuizen

**Affiliations:** ^1^ Epilepsy Research Centre, Department of Medicine University of Melbourne Austin Health Melbourne Victoria Australia; ^2^ Department of Neurology University of Pennsylvania Perelman School of Medicine Philadelphia Pennsylvania USA

**Keywords:** progressive myoclonus epilepsies

## Abstract

Progressive Myoclonus Epilepsy (PME) is a rare epilepsy syndrome characterized by the development of progressively worsening myoclonus, ataxia, and seizures. A molecular diagnosis can now be established in approximately 80% of individuals with PME. Almost fifty genetic causes of PME have now been established, although some remain extremely rare. Herein, we provide a review of clinical phenotypes and genotypes of the more commonly encountered PMEs. Using an illustrative case example, we describe appropriate clinical investigation and therapeutic strategies to guide the management of this often relentlessly progressive and devastating epilepsy syndrome. This manuscript in the Genetic Literacy series maps to Learning Objective 1.2 of the ILAE Curriculum for Epileptology (*Epileptic Disord*. 2019;**21**:129).

**Key points**

- In the evaluation of PME, certain clinical and EEG findings can help narrow the list of differential diagnoses to specific diseases.
- Important PMEs include Unverricht‐Lundborg Disease, Lafora Disease, Neuronal Ceroid Lipofuscinoses, and Myoclonic Epilepsy with Ragged Red Fibers.
- A molecular diagnosis can now be established in approximately 80% of patients with PME. While PME gene panels are currently useful, exome or genome sequencing will likely supplant these in the future.
- Treatment mainly symptomatic.

## 1 PROGRESSIVE MYOCLONUS EPILEPSY

Progressive Myoclonus Epilepsy (PME) is a rare generalized epilepsy syndrome, clinically characterized by the development of progressively worsening myoclonus, ataxia, and tonic–clonic seizures,^1^ with variable associated cognitive decline and neuropsychiatric disturbance.^2^, ^3^ Onset is often in later childhood or adolescence and patients typically become extremely functionally impaired by their symptoms. As the name suggests, myoclonus is core to the PME phenotype. Stimulus‐sensitive myoclonus (also referred to as reflex myoclonus, that is myoclonus induced or exacerbated by a variety of stimuli including light, sound, touch, and action) is a key manifestation. Action‐induced myoclonus is a characteristic feature and is typically fragmentary and multifocal. Myoclonus tends to be refractory to treatment and cause the most functionally significant impairment.^4^

There are many forms of PME, with now almost fifty established genetic causes, although some are extremely rare (see Table S1, ^5^). The most common cause of PME worldwide is Unverricht‐Lundborg Disease (ULD),^6^ with highest reported prevalence in Finland (1.9/100 000).^7^ Other important causes of PME include Lafora Disease (LD, prevalence 1–9/10^6^, ^8^), the Neuronal Ceroid Lipofuscinoses (NCL), and Myoclonic epilepsy with ragged red fibers (MERRF). Geographical distribution varies for some forms of PME, particularly those with recessive inheritance, dependent on the frequency of consanguinity and local founder effects.

## 2 ETIOLOGY AND GENETICS OF PME

A molecular diagnosis can now be established in approximately 80% of patients with PME.^9^, ^10^ Clinical phenotypes and genotypes associated with some of the more common forms of PME are outlined below.

### 2.1 Unverricht‐Lundborg disease

ULD is an autosomal recessive condition due to homozygous or compound heterozygous pathogenic variants in *CSTB*.^6^ The most common type of genetic variant, seen in 90% of disease alleles worldwide, is an unstable expansion of a 12‐nucleotide dodecamer repeat (5′‐CCCCGCCCCGCG‐3′).^11^ ULD‐associated alleles typically contain at least 30 repeat copies.^12^ Although initial small studies did not show a correlation between the expansion size and clinical features, a larger study suggested that the size of the *CSTB* expansion is likely to have a modulating effect on age of disease onset, severity of myoclonus, and neurophysiological markers.^13^ Anticipation (that is increase in expansion from parent to child) has not been identified. Missense pathogenic variants in *CSTB* account for a minority of causative variants.^14^ Individuals who are compound heterozygotes, with one missense and one repeat expansion variant, have been reported to have a more severe disease phenotype.^15^, ^16^

Typical onset of ULD is in late childhood to adolescence (peak 12–13 years of age). Myoclonus is the predominant feature at onset, progressing over months to years before plateauing in middle life (17). Medication refractory reflex myoclonus, particularly action myoclonus, is common and the main cause of functional disability. Other than myoclonus, generalized tonic–clonic seizures (GTCS) are the most common seizure type, with absence and focal seizures with impaired awareness reported rarely.^4^ GTCS typically respond to antiseizure medication, but also independently decrease in frequency as the disease progresses.^17^ The most commonly associated neurological manifestation is ataxia. Cognition is relatively preserved, although subtle impaired processing and executive function have been reported.^18^, ^19^ Psychiatric co‐morbidities are common, with high rates of depression and suicidal behaviour.^18^ Reduced life expectancy is seen, with a median age at death of 53.9 years.^7^

### 2.2 Lafora disease

Lafora Disease is an autosomal recessive condition due to loss‐of‐function variants in *EPM2A* (65%–70% of cases) or *NHLRC1*/*EPM2B*.^20^ No definitive genotype–phenotype correlations have been established, though a longer life expectancy and milder disease course have been described with some *NHLRC1* variants, particularly the recurrent missense variant p.Asp146Asn.^21^, ^22^

*EPM2A* encodes laforin^23^ and *NHLRC1* encodes malin.^24^ Whilst their roles are incompletely understood, both proteins are involved in glycogen metabolism. Evidence suggests they act as a complex to prevent the accumulation of insoluble glycogen.^25^, ^26^, ^27^ LD is associated with the pathognomonic finding of Lafora bodies (diastase‐resistant periodic acid–Schiff (PAS‐D)‐positive aggregates of abnormally branched, insoluble polyglucosans), which are found in the brain, liver, skeletal and cardiac myocytes and sweat glands. It is thought a deficiency of functional laforin or malin results in normally soluble glycogen containing abnormally long chains and precipitating and aggregating as Lafora bodies.^28^

Typical onset of LD is in late childhood or early adolescence (8–19 years, peak at 14–16 years). Focal occipital seizures are a characteristic feature particularly early in disease.^29^ Myoclonus manifests early and escalates rapidly over months to years to intractable action‐ and stimulus‐sensitive myoclonus. Various other seizure types (GTCS, absence, atonic) rapidly develop. LD is associated with a rapidly progressive dementia with significant apraxia, visual loss, and neuropsychiatric disturbance. The prognosis is poor. Early studies suggested death occurred within 10 years of disease onset,^28^, ^30^ but more recent data suggests the prognosis may be less grim, with a median survival of 11 years.^31^ Late onset (>18 years) appears to be related to longer disease duration and slower progression.^31^

### 2.3 Neuronal ceroid lipofuscinoses (NCL)

The NCLs, also known as Batten Disease, are a group of monogenic neurodegenerative conditions grouped together due to a shared histopathological signature, namely abnormal intracellular lysosomal lipopigment storage material with various characteristic forms on ultrastructural examination of brain and peripheral tissues such as skin or lymphocytes. To date, 13 different NCL‐related genes have been identified. Although some clinical heterogeneity is seen, broadly the clinical phenotype includes a combination of seizures, visual failure, dementia, and decline in motor function. Most forms of NCL have onset in infancy or early childhood. Important childhood forms of NCL that can present with a PME phenotype include CLN2 disease, CLN5 disease, and the late‐infantile form of CLN6 disease. CLN2 disease, due to pathogenic variants in *TPP1*, is associated with visual loss and significant language delay.^32^ It is the only form of PME for which there is disease‐modifying treatment, hence prompt recognition and diagnosis is of critical importance (see treatment section below). *KCTD7* pathogenic variants have been described in individuals with a PME phenotype both with and without histopathological evidence of abnormal lysosomal storage material on biopsy.^33^, ^34^

A PME phenotype can also be associated with the recessive adult‐onset NCLs (also referred to as Kufs disease), particularly CLN6 disease. Vision is typically preserved in the adult‐onset NCLs. CLN4 disease, due to pathogenic variants in *DNAJC5*, is the only autosomal dominant form of NCL described, and also presents with an adult‐onset PME phenotype.

### 2.4 Myoclonic epilepsy and ataxia due to KCNC1 mutation (MEAK)

MEAK is a form of PME caused by a recurrent heterozygous missense variant, p.Arg320His in *KCNC1*. Most cases are sporadic due to de novo variants but a few families with autosomal dominant inheritance are reported.^35^ *KCNC1* encodes K_v_3.1, a subunit of the K_v_3 subfamily of voltage‐gated tetrameric potassium channels.^35^ The p.Arg320His variant has a dominant‐negative loss‐of‐function effect *in vitro*.^35^ K_v_3.1 expression is largely restricted to the central nervous system, with predominant expression in GABAergic interneurons.^36^ Thus, disinhibition due to impaired activity of these inhibitory interneurons is a plausible pathogenic mechanism.^35^

The clinical phenotype is characterized by the onset of myoclonus between 6 and 14 years of age.^8^ Myoclonus is the most prominent clinical feature, typically becoming very severe during adolescence and impacting mobility. Infrequent GTCS are seen, and cognition is largely preserved. Early death has not been observed.

### 2.5 Myoclonic epilepsy associated with ragged red fibers (MERRF)

MERRF is one of many mitochondrial disorders due to pathogenic variants in mitochondrial DNA. Mitochondrial DNA encodes polypeptides involved in the mitochondrial respiratory chain. Almost all reported MERRF pathogenic variants affect the gene for tRNA lysine *MT‐TK*, with the m.8344A>G variant identified in 80%–90% of patients.^37^

MERRF is characterized by myoclonus, generalized seizures, myopathy, and progressive ataxia. Onset is typically in childhood, although adult onset is seen. As is typical of mitochondrial disorders, the phenotype is extremely variable, and multisystem involvement (particularly affecting tissues with high metabolic demand) is seen.^38^ Common associated features include hearing impairment, sensorimotor peripheral neuropathy, cognitive impairment, short stature, and optic atrophy. Less common features include cardiomyopathy, diabetes mellitus, pyramidal signs, retinitis pigmentosa, and ophthalmoplegia. Multiple lipomas, ranging from small subcutaneous nodules to large masses, can be seen, typically on the neck or trunk.^38^, ^39^ There is some phenotypic overlap with other mitochondrial disorders, particularly Mitochondrial Encephalopathy, Lactic Acidosis, and Stroke‐like episodes (MELAS). Although acute episodes of neurological impairment are the most common neurological presentation associated with MELAS, a broad range of neurological manifestations including PME are well‐recognized.

### 2.6 Sialidosis

Sialidosis is an autosomal recessive lysosomal storage disorder due to pathogenic variants in *NEU1*, resulting in sialidase deficiency and abnormal accumulation of sialic acid‐rich substrates in the CNS and other organ systems.^40^ There are two forms of sialidosis, type I (normomorphic) and II (dysmorphic). Patients with type I sialidosis have some residual sialidase activity, and exhibit a PME phenotype characterized by progressively worsening multifocal myoclonus with seizures and ataxia. Typical age of onset is between 10 and 20 years. A characteristic macular “cherry‐red spot” can be detected, due to storage material in perifoveal ganglionic cells, and can result in visual impairment.^41^ Early in the disease, the cherry‐red spot may be clinically undetectable, and it may also disappear in the later stages of the disease.^42^

## 3 FURTHER FORMS OF PME

Other well‐described forms of PME include Action Myoclonus Renal Failure Syndrome (AMRF, *SCARB2*), *GOSR2* “North Sea” PME, and Spinomuscular Atrophy‐PME (SMA‐PME, *ASAH1*) amongst others. The most distinguishing (but not universal) feature of AMRF is renal involvement, initially presenting as proteinuria and then progressing to nephrotic syndrome and end‐stage renal failure. “North‐Sea” PME is so‐called as all described cases have birthplaces clustered around the North Sea, a result of a founder effect. A persistently raised CK in the context of a normal muscle biopsy is typical, and associated clinical features include scoliosis and peripheral neuropathy. SMA‐PME is a rare condition characterized by the presence of both lower motor neuron degeneration and classical PME features; either can occur first.^43^ Furthermore, a PME phenotype can also be seen as part of a wider neurological spectrum of disease in various conditions including Dentatorubral pallidoluysian atrophy (DRPLA), Gaucher's Disease, and Juvenile Huntington's Disease.

## 4 RARE AND EMERGING FORMS

With now almost fifty genes associated with PME, many forms remain extremely rare. The biochemical pathways involved are diverse and constantly expanding, as highlighted by the recent novel association of dolichol‐dependent glycosylation with the PME phenotype due to pathogenic variants impacting *NUS1*, *DHDDS*, and *ALG10*.^9^ There are increasing descriptions of cases of PME due to pathogenic variants in genes associated with other neurodevelopmental conditions, broadening the spectrum of clinical phenotypes associated with these genes. This includes but is not limited to the developmental and epileptic encephalopathies (for example, *TBC1D24*, *DHDDS, CHD2, and CACNA2D2)*.

## 5 THE UNSOLVED RESIDUUM

Despite the rapidly evolving understanding of the genetic architecture of PME, 20% of individuals remain without a molecular diagnosis. This unsolved residuum will likely be a collection of ultra‐rare causes in novel genes, or due to pathogenic variants, which are more challenging to identify and interpret with current available technologies, such as repeat expansion mutations, or variants in noncoding regions affecting mechanisms such as transcriptional regulation or epigenetic modification. Nongenetic etiologies, including autoimmune, may warrant consideration, as highlighted by the association of coeliac disease with various neurological manifestations including myoclonus and ataxia.^44^, ^45^

Of note, the Progressive Myoclonic Ataxias (PMAs) are defined as conditions generally presenting first with prominent ataxia, before subsequently developing myoclonus and in some cases epilepsy.^46^ There is clearly a significant clinical phenotypic overlap between PME and PMA, and several conditions are described under both categories in the literature. An awareness of this when considering differential diagnoses and potential underlying etiologies in those who remain without a genetic diagnosis is important.

## 6 INVESTIGATION STRATEGIES

### 6.1 History and examination

PME should be considered in patients with myoclonus (especially if refractory to medication), progressive motor impairment, cognitive deterioration, sensory, and cerebellar signs. Because most genetic causes are recessive, family history may be unrevealing, or may include affected siblings only. Inquiring about consanguinity is essential. Some features of history and examination may suggest specific PME syndromes (Table 1).

**TABLE 1:** History and examination features suggestive of specific PME syndromes.

Finding	Associated syndrome
Visual seizures (history) Occipital seizures (EEG)	Lafora disease
Vision loss, retinopathy	NCLs (but rarely in adult‐onset cases) Sialidosis
Cherry‐red spot in macula	Sialidosis (also Niemann–Pick disease, Gaucher disease)
Myopathy Hearing loss Cutaneous lipomas	MERRF
Stroke‐like attacks	MELAS
Hepatosplenomegaly	Gaucher disease
Peripheral neuropathy Ataxia Optic atrophy Photosensitivity (at low flash rate) Psychiatric symptoms	Multiple PME syndromes, nonspecific

### 6.2 EEG and imaging

EEG should be performed in all patients. It is important to recognize that there can be considerable variability in EEG characteristics between the various PMEs. In most, but not all, forms of PME, the background is typically normal early in the disease course, progressing to irregular diffuse slowing as the disease progresses. Epileptiform discharges are typically generalized spike‐and‐wave or polyspike‐and‐wave, but focal or multifocal epileptiform activity has also been reported in several different PME subtypes.^47^, ^48^ Occipital discharges and/or seizures are often seen in PME, particularly in Lafora disease.^28^ Photosensitivity is common in many of the PMEs.^49^ In particular, photosensitivity at low flash frequencies (<6 Hz) should raise suspicion for a PME, although it is not entirely specific for PME nor for an individual PME subtype.^50^, ^51^ Neuroimaging in patients with PME is typically normal or shows nonspecific atrophy.^52^

### 6.3 Genetic testing

**FIGURE 1:**
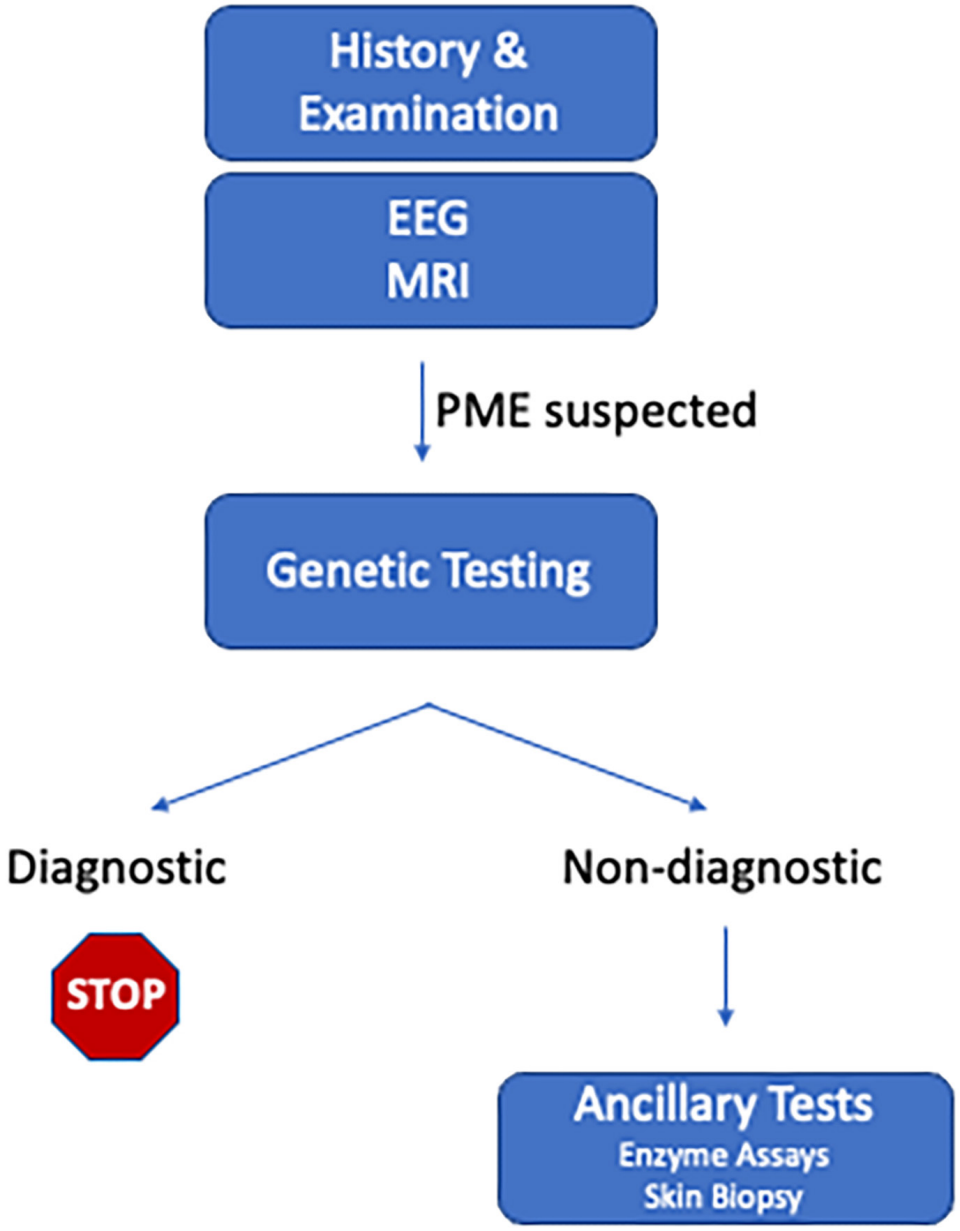
Diagnostic approach to suspected PME.

Genetic testing should now be the first‐line diagnostic test whenever PME is suspected (Figure 1), and should be performed early given the profound prognostic implications of the diagnosis and emerging targeted therapies. Genetic testing for PME is now widely available, noninvasive, and high‐yield. The long and growing list of causative genes (Table S1) warrants a broad testing strategy, rather than targeted single‐gene testing based on phenotype. Gene panels for PME or comprehensive epilepsy panels are available from most commercial laboratories. Exome or genome sequencing have additional advantages and are replacing gene panels as first‐line genetic tests because they can detect findings not detected by a gene panel and enable future reanalysis for undiagnosed cases. Many diagnostic laboratories now offer a gene panel off an exome or genome backbone, which allows for reanalysis with an updated gene list after 18 months–2 years.

Clinicians should be aware of several pitfalls in genetic testing for PMEs. Repeat expansions (as seen in ULD and DRPLA) are typically not captured well by current sequencing‐based tests (including gene panels and exome sequencing) and may require specialized PCR testing or Southern blot, or specific bioinformatics added to genome sequencing to detect expansion variants. Thus the ability of a test to cover these expansion variants should be discussed with the diagnostic laboratory. An additional pitfall of gene panel or exome sequencing is that sequencing of mitochondrial DNA is not standard. It is important that clinicians are aware of this, though typically mitochondrial DNA testing is only appropriate to include as a first‐line genetic test if there are specific clinical features, which suggest an underlying mitochondrial pathology. Whole genome sequencing has the advantage of being able to cover both expansion variants and mitochondrial variants, as well as complex structural and noncoding variants, and so is likely to become the test of choice for PME as costs of WGS come down and availability increases.

Many PMEs are autosomal recessive. This is important when interpreting the results of genetic testing. For recessive disorders, a single heterozygous pathogenic variant is not sufficient to cause disease; such a patient is an unaffected carrier. On the other hand, when a particular syndrome is strongly suspected but only a single heterozygous pathogenic variant is detected, an occult second pathogenic variant may exist, but not yet have been detected by the testing which has been ordered to date.^53^ This means that broadening the testing, for example, to whole genome sequencing, which can detect a wider variety of genomic mechanisms, may be appropriate, and discussion with a clinical genetics team can be helpful to discuss the next best steps. When two different pathogenic variants are identified in the same gene (compound heterozygous), it is necessary to confirm that the two variants are on different alleles (“in trans”), that is one inherited from each parent, rather than on the same allele (“in cis”). This can be checked by segregating the variants in both parents, with the anticipation that each will carry one of the two variants found in the patient, although very occasionally one variant may be de novo and not inherited. Finally, variants of uncertain significance (VUS) must be interpreted with caution, and in most cases should not be considered diagnostic nor used to guide management. Discussion with a genetics team can be helpful to consider if further options, such as functional studies, may be possible to help further investigate VUS. Reanalysis after time with updated knowledge and gene lists is also helpful for families without a confirmed molecular diagnosis.

### 6.4 Ancillary tests

In the past, the evaluation of suspected PME often included an assortment of specialized tests, such as enzyme assays and tissue biopsies. In the modern genetic era, these should be considered ancillary, and may not be necessary If genetic testing is diagnostic. Ancillary testing still plays a role when an orthogonal test may be helpful to support a genetic diagnosis, for example, to help determine the significance of a VUS.

#### 6.4.1 Laboratory tests

Some common laboratory tests may suggest particular PME syndromes. Renal impairment is often a feature of AMRF.^54^ Elevated CK has been associated with North‐Sea PME, despite normal muscle biopsies.^55^ Elevated lactate may suggest a mitochondrial disorder, but is neither sensitive nor specific.

Enzyme assays have been developed for several of the NCLs caused by lysosomal enzyme deficiencies.^56^, ^57^, ^58^ Urine sialo‐oligosaccharides are elevated in sialidosis. These assays are ordered and performed individually, and systematic approaches to the selection of tests are limited.^58^

#### 6.4.2 Neurophysiology

Giant evoked potentials in response to both visual and somatosensory stimulation have been reported in several PMEs.^48^, ^59^, ^60^ Although the diagnostic utility of this finding has not been studied systematically, this is an accessible and noninvasive test, which can be used to demonstrate cortical hyperexcitability in individuals with PME. Nerve conduction and EMG studies may be indicated to evaluate specific comorbid features such as neuropathy or myopathy.

#### 6.4.3 Tissue biopsies

Before the era of readily available molecular testing, tissue‐based diagnosis was a standard part of the diagnostic evaluation of PME.^57^, ^61^ Today, biopsies are not necessary in cases with a clear genetic diagnosis and compatible phenotype. Biopsies may be useful in selected patients with nondiagnostic genetic testing. Tissue diagnosis pertains mostly to LD and the NCLs. The pathognomonic Lafora bodies seen in LD can be identified on axillary skin biopsy, though the sensitivity and specificity are not known, and are likely to vary across institutions based on local experience.

Similarly, the sensitivity and specificity of biopsy for NCL are unknown. In adult‐onset NCL, intracellular storage material may be absent in peripheral tissues (false negatives), while normal accumulation of age‐related lipofuscin may be misinterpreted as pathological (false positives).^62^ When a mitochondrial disorder is suspected, muscle biopsy may show ragged red fibers. Again, the sensitivity and specificity of this test are unknown, and muscle biopsy is now primarily reserved for patients with nondiagnostic genetic testing.

## 7 TREATMENT STRATEGIES IN PME

Management of patients with PME presents several challenges. For most types of PME, there are no available disease‐modifying therapies and treatment strategies are symptomatic, aimed at seizure control and suppression of myoclonus. The rarity of the condition means evidence‐based efficacy data is minimal, and is largely in small populations of ULD or LD if at all.

Antiseizure medications (ASMs) are the current mainstay of treatment. Valproate was the initial ASM shown to be effective in convulsive seizure and myoclonus control in ULD.^63^ Benzodiazepines, including clonazepam and clobazam, have well‐established efficacy for the management of myoclonus, and are typically used as an add‐on therapy. More recently several ASMs including levetiracetam, topiramate, zonisamide, and brivaracetam have been reported to be effective in PME. Piracetam is a useful antimyoclonic agent,^64^ and perampanel has been associated with a beneficial effect on action myoclonus, disability, and seizures in PME.^40^

### 7.1 Adjunctive therapies

Even when used in combination, ASMs typically fail to adequately control the complex and progressive symptomatology experienced by patients with PME, and consideration needs to be given to adjunctive therapies. There is limited data exploring dietary therapeutic strategies in PME, with only small case series of the ketogenic diet and modified Atkins diet in LD and North Sea PME, respectively, showing some improvement in myoclonus.^65^, ^66^ Similarly, limited case reports and case series of neuromodulatory therapies including VNS, DBS and rTMS have variable results, and all warrant further exploration. With the increasing appreciation of the role of neuroinflammation in neurodegenerative disorders, immunomodulatory medications may also have a therapeutic role, though this is yet to be established.^67^, ^68^ Animal data suggests that metformin may have a disease‐modifying role in Lafora disease,^69^, ^70^ though the very limited clinical data that is currently available suggests that its use is not associated with clinically meaningful benefit.^71^ Further data is required to clarify this.

### 7.2 Enzyme replacement therapies

Enzyme replacement is currently available for treatment of CLN2 disease, caused by pathogenic variants in the gene *TPP1*, leading to a deficiency in the lysosomal enzyme tripeptidyl peptidase 1. Enzyme replacement therapy via intraventricular infusion of recombinant human tripeptidyl peptidase 1 (cerliponase alfa) results in less decline in motor and language function that in historical controls.^57^ This is the first disease‐modifying treatment for a type of PME and holds the promise that similar approaches may be successful in other forms of PME. Several PME‐causing pathogenic variants result in enzyme deficiencies (including Sialidosis, SMA‐PME, and Gaucher's disease), and may be good targets for enzyme replacement therapies.

### 7.3 Gene therapies

Gene and nucleotide‐based therapies are not yet clinically available for any of the PMEs. In mouse models of CLN6 disease, bilateral intracerebroventricular injections of adeno‐associated viral 6 carrying functional copies of *CLN6* have been shown to increase lifespan and reduce neuropathological features.^72^ Antisense oligonucleotide (ASO) therapy has been shown both in vitro and in vivo to have beneficial impacts. Patient‐derived fibroblasts from an individual with ULD homozygous for the c.66G>A *CSTB* pathogenic variant expressed normal CSTB protein levels when treated with a specific locked nucleic acid ASO targeting the cryptic donor splice site in intron 1 of *CSTB*.^73^ Such precision medicine therapeutic strategies are clearly still in their initial stages but hold promise that future improved targeted therapies will be feasible for patients with PME.

## Supporting information


Appendix S1.

Data S1.

Table S1.

## APPENDIX 1

### Full list of ILAE Genetics Commission members

Piero Perucca, Bladin‐Berkovic Comprehensive Epilepsy Program, Austin Health, Australia, and Department of Medicine (Austin Health), Melbourne Medical School, The University of Melbourne, Australia; J. Helen Cross, UCL‐Institute of Child Health, Great Ormond Street Hospital for Children, London & Young Epilepsy, Lingfield, UK; Holger Lerche, Department of Neurology and Epileptology, Hertie Institute for Clinical Brain Research, University of Tuebingen, Tuebingen, Germany; Alina I. Esterhuizen, UCT/MRC Genomic and Precision Medicine Research Unit, Division of Human Genetics, Department of Pathology, Institute of Infectious Disease and Molecular Medicine, Faculty of Health Sciences, University of Cape Town, Cape Town, South Africa; Iscia Lopes‐Cendes, Department of Translational Medicine, University of Campinas, Campinas, Brazil; Meng‐Han Tsai, Department of Neurology, Kaohsiung Chang Gung Memorial Hospital and Chang Gung University College of Medicine, Kaohsiung, Taiwan; Daniel H. Lowenstein, Department of Neurology, University of California, San Francisco, USA; Nigel C. K. Tan, Department of Neurology, National Neuroscience Institute, Singapore, Singapore; Ingo Helbig, Division of Neurology, The Children's Hospital of Philadelphia, Philadelphia, USA; Heather C. Mefford, Center for Pediatric Neurological Disease Research, St. Jude Children's Research Hospital, Memphis, USA; Andreas Brunklaus, Institute of Health and Wellbeing, University of Glasgow, UK; Gaetan Lesca, Department of Genetics, Hospices Civils de Lyon, Bron, France.

### Full list of ILAE Genetic Literacy Task Force members

Elizabeth Emma Palmer, Centre of Clinical Genetics. Sydney Children's Hospitals Network, and University of New South Wales, Randwick, NSW, Australia; Amy McTague, Developmental Neurosciences, UCL Great Ormond Street Institute of Child Health, and Department of Neurology, Great Ormond Street Hospital for Children, London, UK; Faiza Fakhfakh, Laboratory of molecular and functional genetics, Faculty of science, Sfax University of Sfax, Tunisia; Norman Delanty, Department of Neurology, Beaumont Hospital, Dublin, and FutureNeuro Research Centre, RCSI, Dublin; Daniel H. Lowenstein, Department of Neurology, University of California, San Francisco, USA; Nigel C. K. Tan, Department of Neurology, National Neuroscience Institute, Singapore, Singapore; Ingo Helbig, Division of Neurology, The Children's Hospital of Philadelphia, Philadelphia, USA; Alina I. Esterhuizen, UCT/MRC Genomic and Precision Medicine Research Unit, Division of Human Genetics, Department of Pathology, Institute of Infectious Disease and Molecular Medicine, Faculty of Health Sciences, University of Cape Town, Cape Town, South Africa.

## APPENDIX 2

### Case study

A 12‐year‐old female was referred to neurology outpatients with a six‐month history of increasing upper and lower limb jerks, which were starting to impact her ability to complete schoolwork. She had a history of three generalized tonic–clonic seizures over a two‐year period, on the background of unremarkable birth and developmental milestones. There was no significant family history. Examination revealed some subtle difficulty with tandem gait, with no other focal neurological deficit. The remainder of her general physical examination was unremarkable. EEG revealed normal background rhythm, with rare generalized spike–wave discharges and a grade IV photoparoxysmal response. A diagnosis of Juvenile Myoclonic Epilepsy was made, and the patient was commenced on 500 mg bd levetiracetam. Over the subsequent 12 months, she had no further convulsive seizures, though she had increasingly severe and frequent upper and lower limb jerks, was quite clumsy, and had several falls despite adjustments to her antiseizure medication including uptitration of levetiracetam. Examination revealed action‐ and stimulus‐sensitive myoclonus and appendicular and truncal ataxia. Further investigations including MRI Brain and basic biochemical testing provided no further diagnostic clues. Cognitive function remained intact. Given the progressive nature of her condition, the diagnosis of Progressive Myoclonic Epilepsy (PME) was considered. After appropriate patient and family genetic counseling, a targeted PME gene panel was requested.

### Case resolution

The targeted PME gene panel did not reveal any pathogenic variants for our patient. CSTB repeat expansion testing was subsequently requested, revealing a homozygous 12‐nucleotide dodecamer repeat expansion, thus providing molecular confirmation of a diagnosis of Unverricht‐Lundborg Disease. Myoclonus continued to progress over the next 3 years, despite the addition of sodium valproate, piracetam, and clonazepam. A wheelchair was required for mobilization at the age of 30 years due to the severity of myoclonus. Test yourself

1. In a 15‐year‐old male with a history of myoclonus and epilepsy, which of the following features might suggest he has a PME?
  - Macular cherry‐red spot
  - Occipital seizures in his EEG
  - Hepatosplenomegaly
  - All of the above
2. The Progressive Myoclonic Epilepsies
  - Are very rarely associated with ataxia
  - Typically present in the 4th and 5th decades of life
  - Present with myoclonus that is fragmentary and multifocal
  - Can be diagnosed using genetic testing in about 25% of cases

*Answers may be found in the* *supporting information*.
