# Supplementary material for: ILAE Genetics Literacy series: Progressive myoclonus epilepsies
Source: Epileptic Disord. 2023 Sep 6;25(5):670–80. doi: 10.1002/epd2.20152 (PMC10947580; doi:10.1002/epd2.20152)
Supplement: Supplementary file 1 — Appendix S1. [file EPD2-25-670-s002.docx]

**TEST YOURSELF**

**Answers:**

1. D

2. C.
