# Supplementary material for: ILAE Genetics Literacy series: Progressive myoclonus epilepsies
Source: Epileptic Disord. 2023 Sep 6;25(5):670–80. doi: 10.1002/epd2.20152 (PMC10947580; doi:10.1002/epd2.20152)
Supplement: Supplementary file 2 — Data S1. [file EPD2-25-670-s003.pptx]

## Slide 1
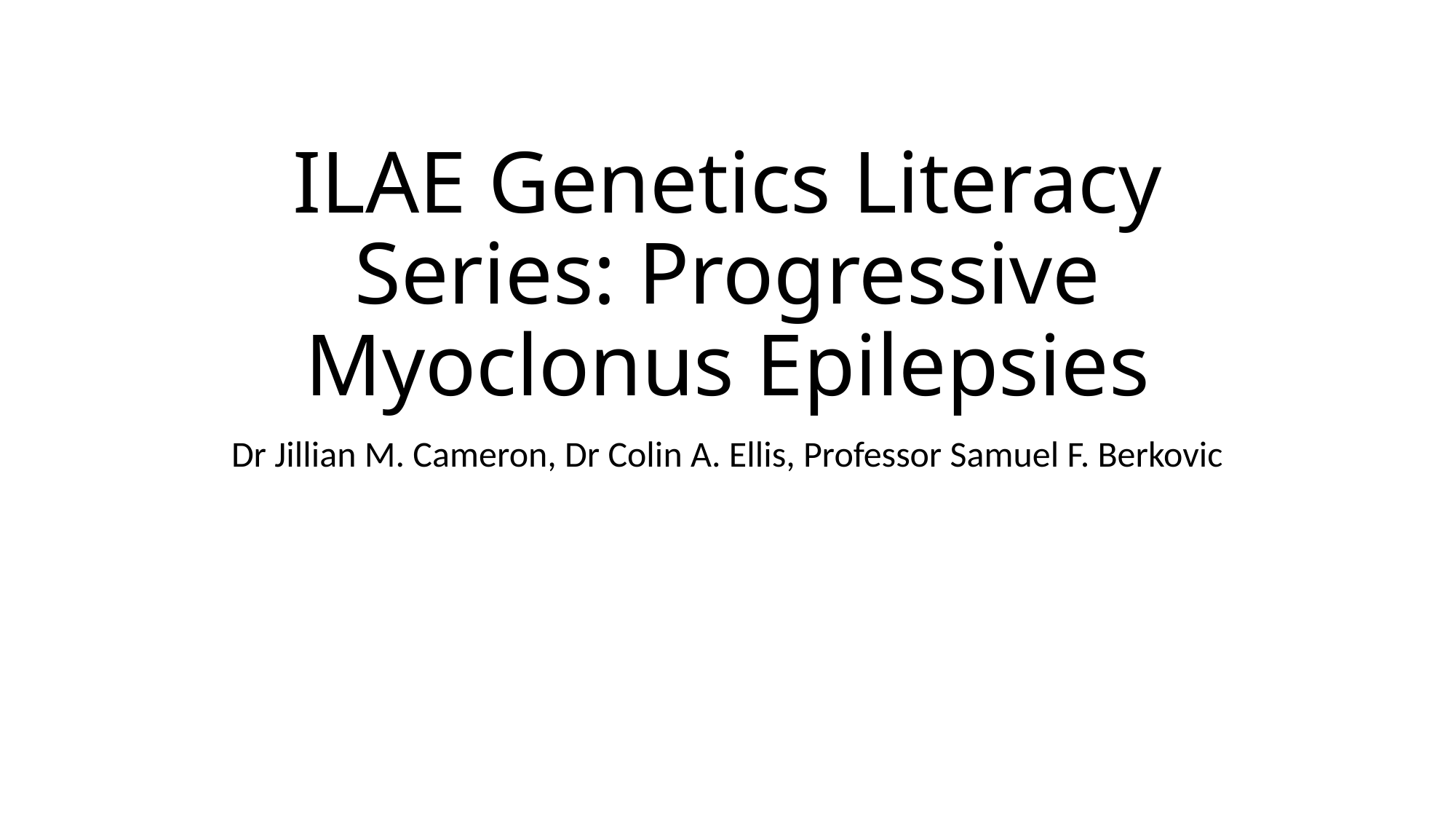

# ILAE Genetics Literacy Series: Progressive Myoclonus Epilepsies
Dr Jillian M. Cameron, Dr Colin A. Ellis, Professor Samuel F. Berkovic

## Slide 2
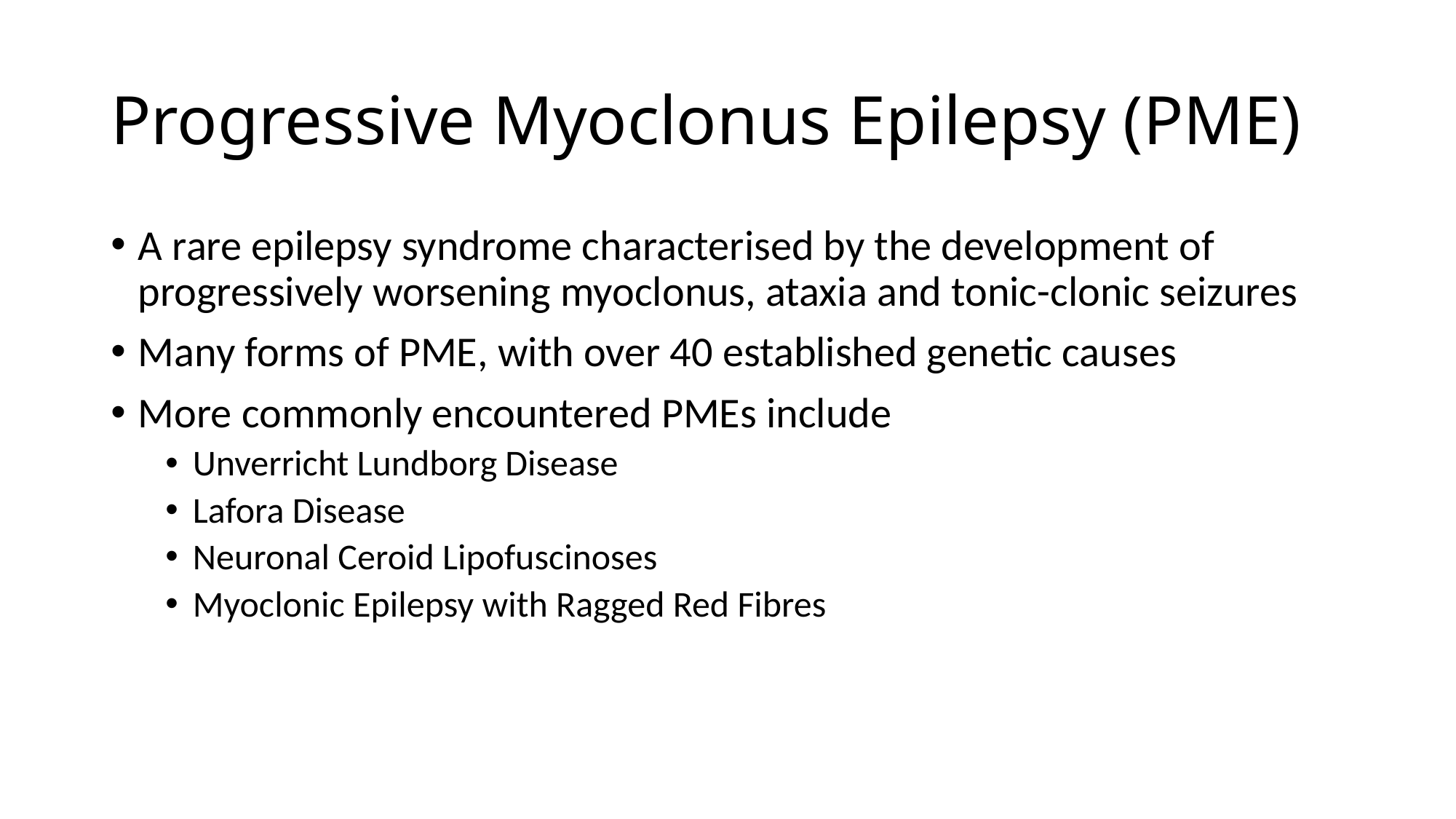

# Progressive Myoclonus Epilepsy (PME)
A rare epilepsy syndrome characterised by the development of progressively worsening myoclonus, ataxia and tonic-clonic seizures
Many forms of PME, with over 40 established genetic causes
More commonly encountered PMEs include
Unverricht Lundborg Disease
Lafora Disease
Neuronal Ceroid Lipofuscinoses
Myoclonic Epilepsy with Ragged Red Fibres

## Slide 3
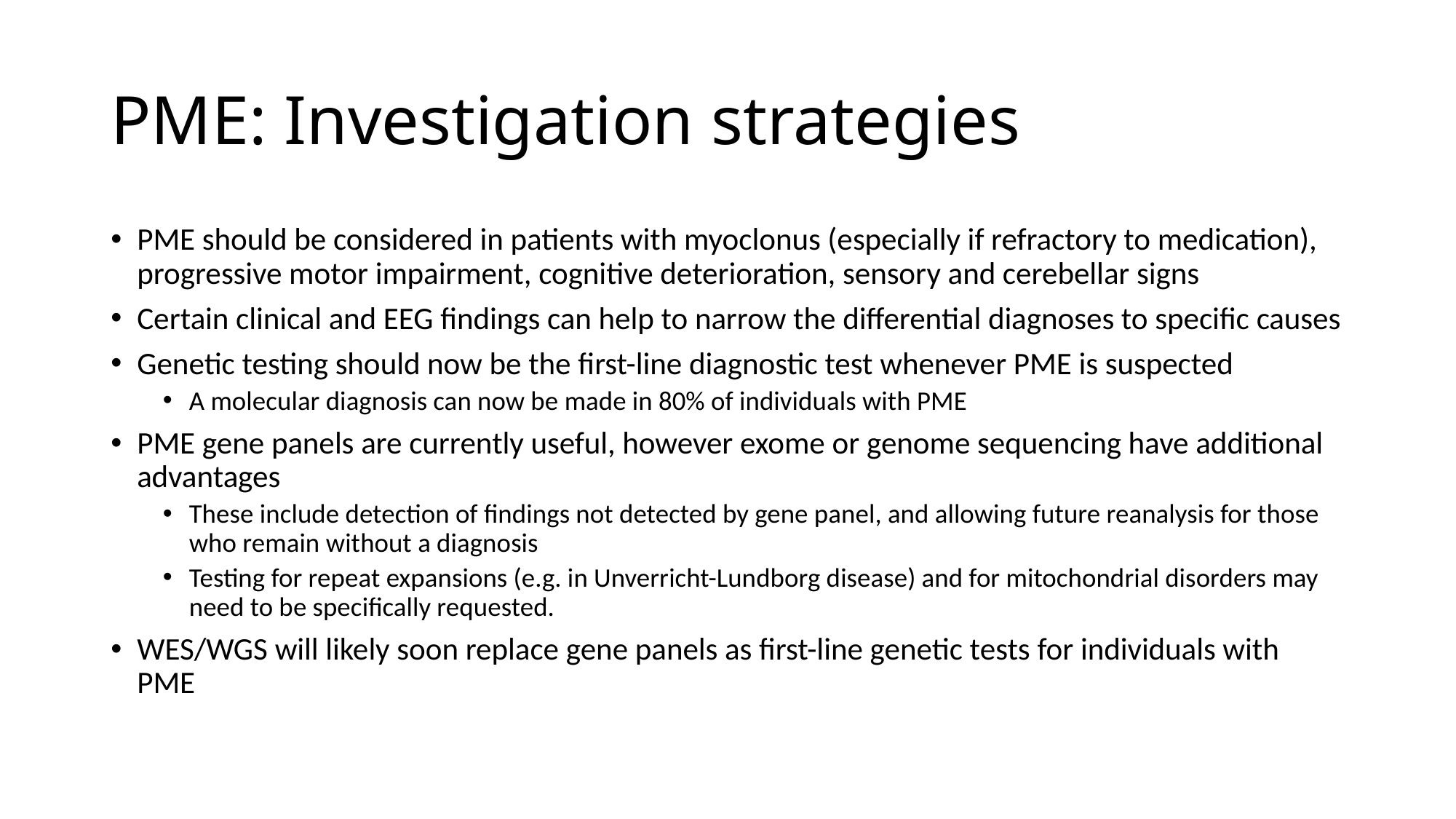

# PME: Investigation strategies
PME should be considered in patients with myoclonus (especially if refractory to medication), progressive motor impairment, cognitive deterioration, sensory and cerebellar signs
Certain clinical and EEG findings can help to narrow the differential diagnoses to specific causes
Genetic testing should now be the first-line diagnostic test whenever PME is suspected
A molecular diagnosis can now be made in 80% of individuals with PME
PME gene panels are currently useful, however exome or genome sequencing have additional advantages
These include detection of findings not detected by gene panel, and allowing future reanalysis for those who remain without a diagnosis
Testing for repeat expansions (e.g. in Unverricht-Lundborg disease) and for mitochondrial disorders may need to be specifically requested.
WES/WGS will likely soon replace gene panels as first-line genetic tests for individuals with PME

## Slide 4
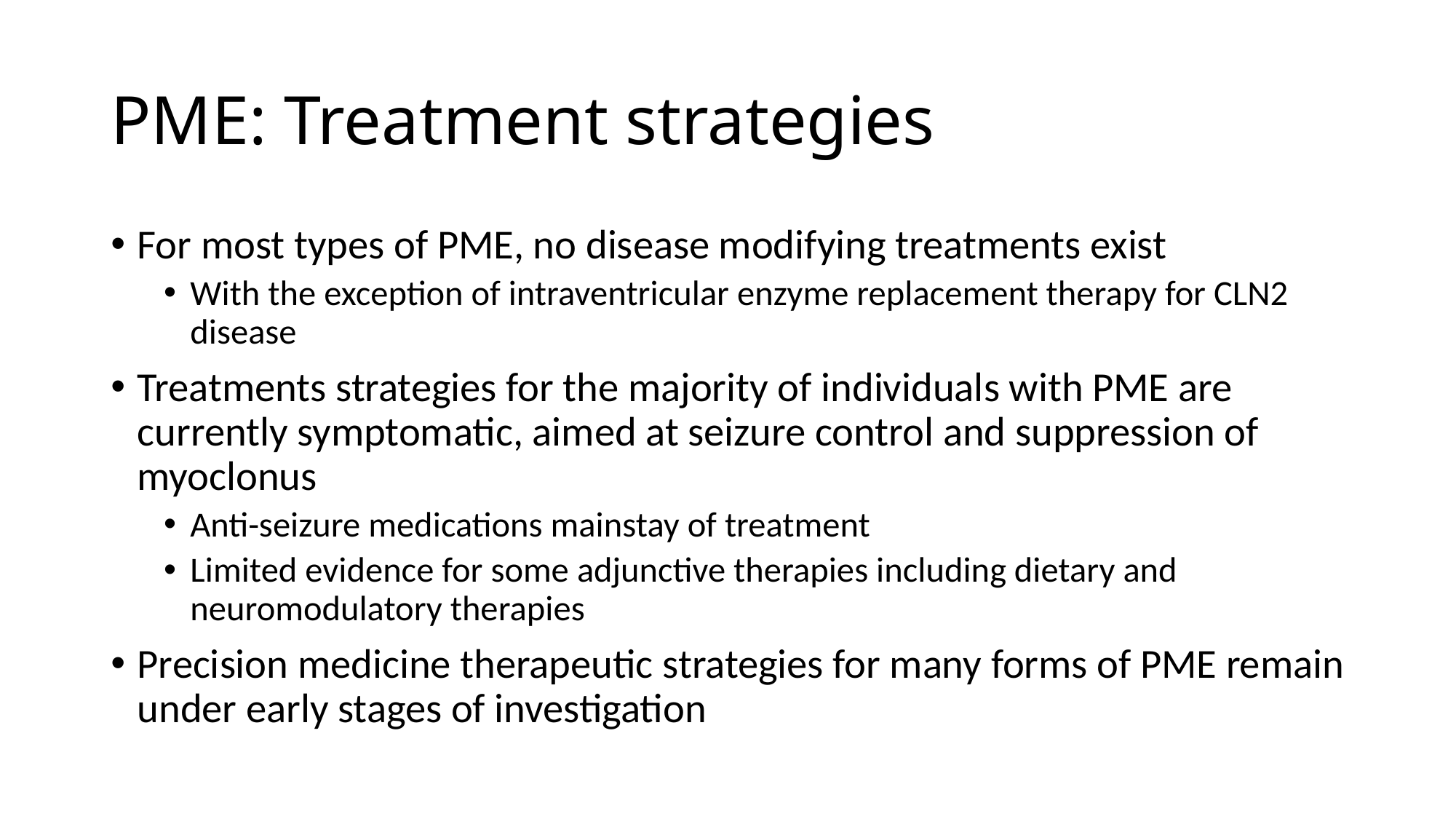

# PME: Treatment strategies
For most types of PME, no disease modifying treatments exist
With the exception of intraventricular enzyme replacement therapy for CLN2 disease
Treatments strategies for the majority of individuals with PME are currently symptomatic, aimed at seizure control and suppression of myoclonus
Anti-seizure medications mainstay of treatment
Limited evidence for some adjunctive therapies including dietary and neuromodulatory therapies
Precision medicine therapeutic strategies for many forms of PME remain under early stages of investigation
