# Supplementary material for: ILAE Genetics Literacy series: Progressive myoclonus epilepsies
Source: Epileptic Disord. 2023 Sep 6;25(5):670–80. doi: 10.1002/epd2.20152 (PMC10947580; doi:10.1002/epd2.20152)
Supplement: Supplementary file 3 — Table S1. [file EPD2-25-670-s001.docx]

| **PME Genes with >20 reported cases in literature** | | | |
| --- | --- | --- | --- |
|  | **Gene** | **Inheritance** | **Disease name** |
|  | *CSTB* | AR | Unverricht-Lundborg Disease |
|  | *EPM2A* | AR | Lafora Disease |
|  | *NHLRC1* | AR | Lafora Disease |
|  | *TPP1* | AR | Neuronal Ceroid Lipofuscinosis (CLN2 Disease) |
|  | *DNAJC5 (CLN4)* | AD | Neuronal Ceroid Lipofuscinosis (CLN4 Disease) |
|  | *CLN5* | AR | Neuronal Ceroid Lipofuscinosis (CLN5 Disease) |
|  | *CLN6* | AR | Neuronal Ceroid Lipofuscinosis (CLN6 Disease) |
|  | *SCARB2* | AR | Action Myoclonus Renal Failure Syndrome (AMRF) |
|  | *KCNC1* | AD | Myoclonic Epilepsy with Ataxia due to potassium channel mutation (MEAK) |
|  | *GBA* | AR | Gaucher Disease |
|  | *GOSR2* | AR | North Sea PME |
|  | *ATN1* | AD | Dentatorubral-pallidoluysian atrophy (DRPLA) |
|  | *KCTD7* | AR |  |
|  | *NEU1* | AR | Sialidosis |
|  | *MT-TK* | MT | Myoclonus Epilepsy with Ragged Red Fibers (MERRF) |
|  | *ASAH1* | AR | Spinal muscular atrophy with PME (SMA-PME) |
|  | *HTT* | AD | Juvenile Huntington’s Disease |
| **Rare PME Genes** | | | |
|  | *AFG3L2* | AR |  |
|  | *ALG10* | AR |  |
|  | *CERS1* | AR |  |
|  | *GRN* | AR |  |
|  | *IRF2BPL* | AD |  |
|  | *LMNB2* | AR |  |
|  | *NUS1* | AD |  |
|  | *SEMA6B* | AD |  |
|  | *SERPINI1* | AD | Neuroserpinopathy |
|  | *SLC7A6OS* | AR |  |
|  | *PRDM8* | AR |  |
| **Genes usually associated with other phenotypes, with PME phenotype reported** | | | |
|  | *MT-TL1* | MT | Mitochondrial Encephalopathy, Lactic Acidosis and Stroke-like Episodes (MELAS) |
|  | *ATP13A2* | AR |  |
|  | *CHD2* | AD |  |
|  | *SCN8A* | AD |  |
|  | *TBC1D24* | AR |  |
|  | *ATP6V0A1* | AD/AR |  |
|  | *CACNA2D2* | AR |  |
|  | *DHDDS* | AD |  |
|  | *KCNA2* | AD |  |
|  | *FBX028* | AD |  |
|  | *CACNA1A* | AD |  |
|  | *SACS* | AR |  |
|  | *STUB1* | AR |  |
|  | *NAXE* | AR |  |
|  | *POLG* | AR |  |
|  | *RARS2* | AR |  |
|  | *MT-ATP6* | MT |  |
|  | *PEX19* | AR |  |
|  | *PRNP* | AD |  |

**Supplementary Table 1:** Genes associated with PME phenotypes
